# Supplementary material for: Cancer type classification using plasma cell-free RNAs derived from human and microbes
Source: eLife. 2022 Jul 11;11:e75181. doi: 10.7554/eLife.75181 (PMC9273212; doi:10.7554/eLife.75181)
Supplement: Supplementary file 1. [file elife-75181-supp1.docx]

**Table S1. Clinical characteristic of the study participants**

| **Healthy Donor**  **(n=76)** | | | | | **Colorectal Cancer**  **(n=54)** | | | | **Stomach Cancer**  **(n=37)** | | | | | | | **Liver Cancer**  **(n=62)** | | | | | | **Lung Cancer**  **(n=35)** | | | | | | | **Esophageal Cancer**  **(n=31)** | | | |  |
| --- | --- | --- | --- | --- | --- | --- | --- | --- | --- | --- | --- | --- | --- | --- | --- | --- | --- | --- | --- | --- | --- | --- | --- | --- | --- | --- | --- | --- | --- | --- | --- | --- | --- |
| **Variable** | **No.** | | **%** | | **Variable** | **No.** | | **%** | **Variable** | | **No.** | | | | **%** | **Variable** | | **No.** | | | **%** | **Variable** | | **No.** | | | | **%** | **Variable** | **No.** | **%** | |  |
| Age, years | |  |  | | Age, years |  | |  | Age, years | |  | | | |  | Age, years | |  | | |  | Age, years | | | |  | |  | Age, years |  |  | |  |
| Mean | 59 | |  | | Mean | 65 | |  | Mean | | 63 | | | |  | Mean | | 57 | | |  | Mean | | 62 | | | |  | Mean | 64 |  | |  |
| SD | 11 | |  | | SD | 11 | |  | SD | | 9 | | | |  | SD | | 11 | | |  | SD | | 8 | | | |  | SD | 10 |  | |  |
| Sex |  | |  | | Sex |  | |  | Sex | |  | | | |  | Sex | |  | | |  | Sex | |  | | | |  | Sex |  |  | |  |
| Male | 40 | | 53% | | Male | 36 | | 67% | Male | | 30 | | | | 81% | Male | | 50 | | | 81% | Male | | 17 | | | | 49% | Male | 29 | 94% | |  |
| Female | 36 | | 47% | | Female | 18 | | 33% | Female | | 7 | | | | 19% | Female | | 12 | | | 19% | Female | | 18 | | | | 51% | Female | 2 | 6% | |  |
|  |  | |  | | Stage |  | |  | Stage | |  | | | |  | Stage | |  | | |  | Stage | |  | | | |  | Stage |  |  | |  |
|  |  | | |  | 1 | 6 | | 11% | 1 | | 15 | | | | 41% | 0 | | 4 | | | 6% | 1 | | 24 | | | | 69% | 1 | 14 | 45% | |  |
|  |  | | |  | 2 | 39 | | 72% | 2 | | 9 | | | | 24% | A | | 40 | | | 65% | 2 | | 6 | | | | 17% | 2 | 8 | 26% | |  |
|  |  | | |  | 3 | 9 | | 17% | 3 | | 11 | | | | 30% | B | | 6 | | | 10% | 3 | | 5 | | | | 14% | 3 | 9 | 29% | |  |
|  |  | | |  | 4 | 0 | | 0 | 4 | | 2 | | | | 5% | C | | 7 | | | 11% | 4 | | 0 | | | | 0 | 4 | 0 | 0 | |  |
|  |  | | |  |  |  | |  |  | |  | | | |  | Unknown | | 5 | | | 8% |  | |  | | | |  |  |  |  | |  |
|  |  | | |  | MMR |  | |  | HER2 | |  | | | |  | CHB^c^ history | | | | |  | Smoking history | | | | | |  |  |  |  | |  |
|  |  | | |  | Deficient | 0 | | 0 | Positive | | 13 | | | | 35% | Yes | | 37 | | | 60% | Yes | | 7 | | | | 20% |  |  |  | |  |
|  |  | | |  | Normal | 50 | | 93% | Negative | | 20 | | | | 54% | No | | 22 | | | 35% | No | | 28 | | | | 80% |  |  |  | |  |
|  |  | | |  | No biopsy | 4 | | 7% | No biopsy | | 4 | | | | 11% | Others | | 3 | | | 5% |  | |  | | | |  |  |  |  | |  |
|  |  | | |  | Tumor size |  | |  | Tumor size | | | | |  |  | Tumor size | |  | | |  | Tumor size | | | |  | |  | Tumor size |  |  | |  |
|  |  | | |  | ≤ 3 cm | 12 | | 22% | ≤ 3 cm | | 16 | | | | 43% | ≤ 3 cm | 12 | | | | 19% | No biopsy | | | 35 | | | 100% | No biopsy .31 | | 100% | |  |
|  |  | | |  | > 3 cm | 42 | | 78% | > 3 cm | | 19 | | | | 51% | > 3 cm | | 50 | | | 81% |  | |  | | | |  |  | |  | |  |
|  |  | | |  |  |  | |  | No biopsy | | 2 | | | | 6% |  | |  | | |  |  | |  | | | |  |  | |  | |  |
|  |  | | |  | Vascular Invasion | | |  | Vascular Invasion | | | | | |  | Vascular Invasion | | | | |  | Vascular Invasion | | | | | |  | Vascular Invasion | |  | |  |
|  |  | | |  | Yes | 11 | | 20% | Yes | | 10 | | | | 27% | Yes | | 2 | | | 3% | Yes | | 3 | | | | 9% | Yes | 6 | 19% | |  |
|  |  | | |  | No | 43 | | 80% | No | | 25 | | | | 68% | No | | 36 | | | 58% | No | | 26 | | | | 74% | No | 14 | 45% | |  |
|  |  | | |  |  |  | |  | No biopsy | | 2 | | | | 5% | No biopsy | | 24 | | | 39% | No biopsy | | 6 | | | | 17% | No biopsy | 11 | 36% | |  |
|  |  | | |  | Category | | |  | Category | | | | | |  | AFP | | | | |  | Category | | | | | |  | Category | | |  |  |
|  |  | | |  | Adenocarcinoma 54 | | | 100% | Adenocarcinoma 37 | | | | | | 100% | ≤400ng/ml | | | 49 | 79% | | NSCLC^d^ | ,35 | | | | 100% | | Squamous cell 21 | | 68% | |  |
|  |  | | |  | Others 0 | | | 0 | Others | | | 0 | | | 0 | > 400ng/ml | | | 13 | 21% | | Others | | .0 | | | | 0 | No biopsy | 10 | 32% | |  |
|  |  | | |  | Tumor Site | | |  | Tumor Site | | | | | |  | Category | |  | | |  |  | | | | | | | Tumor Site |  |  | |  |
|  |  | | |  | Rectum | | 21 | 39% | Antrum | | 18 | | | | 49% | HCC^e^ | | 52 | | | 84% |  | | | | | | | Upper | 2 | 7% | |  |
|  |  | | |  | Sigmoid colon | | 14 | 26% | Fundus | | 9 | | | | 24% | ICC^f^ | | 8 | | | 13% |  | |  | | | |  | Middle | 11 | 35% | |  |
|  |  | | |  | Ascending colon | | 8 | 15% | Others | 10 | | | | | 27% | HCC-ICC | | 2 | | | 3% |  | |  | | | |  | Bottom | 8 | 26% | |  |
|  |  | | |  | Transverse colon | | 2 | 4% |  | | | |  | |  |  | |  | | |  |  | |  | | | |  | No biopsy. .10 | | 32% | |  |
|  |  | | |  | Descending colon | | 3 | 5% |  | | | |  | |  |  | |  | | |  |  | |  | | | |  |  | |  | |  |
|  |  | | |  | Others | | 6 | 11% |  | | | |  | |  |  | |  | | |  |  | |  | | | |  |  | |  | |  |
| Clinical Center | | | |  | Clinical Center | | |  | Clinical Center | | | | | |  | Clinical Center | | | | |  | Clinical Center | | | | | |  | Clinical Center | |  | |  |
| PKU^1^ | 19 | | 25% | | PKU | | 54 | 100% | PKU | | | | 37 | | 100% | ShH-2^4^ | | 38 | | | 61% | PKU | | 35 | | | | 100% | PKU | 23 | 74% | |  |
| ShH-1^2^ | 36 | | 47% | |  | |  |  |  | | | |  | |  | ShH-3^5^ | | 16 | | | 26% |  | |  | | | |  | PUMCH^6^ | 8 | 26% | |  |
| SWU^3^ | 21 | | 28% | |  | |  |  |  | | | |  | |  | SWU | | 8 | | | 13% |  | |  | | | |  |  | |  | |  |
| ^a^MMR: Mismatch repair gene; ^b^HER2: Human epidermal growth factor receptor 2; ^c^CHB: Chronic Hepatitis B; ^d^NSCLC: Non-small cell lung cancer; ^e^HCC: Hepatocellular carcinoma; ^f^ICC: Intrahepatic cholangiocarcinoma.   1. ^1^PKU: Peking University First Hospital, Beijing, China; ^2^ShH-1: Department of Epidemiology, Navy Medical University, Shanghai, China; ^3^SWU: Southwest Hospital, Chongqing, China; ^4^ShH-2: Eastern Hepatobiliary Surgery Hospital, Shanghai, China; ^5^ShH-3: National Center for Liver Cancer, Shanghai, China; ^6^PUMCH: Peking Union Medical College Hospital, Beijing, China. | | | | | | | | | | | | | | | | | | | | | | | | | | | | | | | | |  |
